# Monophyly of the species of *Hepatozoon* (Adeleorina: Hepatozoidae) parasitizing (African) anurans, with the description of three new species from hyperoliid frogs in South Africa

**DOI:** 10.1017/S003118201700213X

**Published:** 2017-12-04

**Authors:** Edward C. Netherlands, Courtney A. Cook, Louis H. Du Preez, Maarten P.M. Vanhove, Luc Brendonck, Nico J. Smit

**Affiliations:** 1 Water Research Group, Unit for Environmental Sciences and Management, North-West University, Private Bag X6001, Potchefstroom 2520, South Africa; 2 African Amphibian Conservation Research Group, Unit for Environmental Sciences and Management, North-West University, Private Bag X6001, Potchefstroom 2520, South Africa; 3 Laboratory of Aquatic Ecology, Evolution and Conservation, University of Leuven, Charles Debériotstraat 32, Leuven B-3000, Belgium; 4 Department of Zoology and Entomology, University of the Free State, QwaQwa campus, Free State, South Africa; 5 South African Institute for Aquatic Biodiversity, Somerset Street, Grahamstown 6140, South Africa; 6 Capacities for Biodiversity and Sustainable Development, Operational Directorate Natural Environment, Royal Belgian Institute of Natural Sciences, Vautierstraat 29, Brussels B-1000, Belgium; 7 Laboratory of Biodiversity and Evolutionary Genomics, University of Leuven, Charles Debériotstraat 32, Leuven B-3000, Belgium; 8 Centre for Environmental Sciences, Research Group Zoology: Biodiversity & Toxicology, Hasselt University, Agoralaan Gebouw D, Diepenbeek B-3590, Belgium; 9 Department of Botany and Zoology, Faculty of Science, Masaryk University, Kotlářská 2, Brno CZ-611 37, Czech Republic

**Keywords:** *Afrixalus*, amphibia, apicomplexan, blood parasite, haemogregarine, Hyperoliidae, *Hyperolius*, morphology, phylogenetic analysis

## Abstract

Haemogregarines (Apicomplexa: Adeleiorina) are a diverse group of haemoparasites reported from almost all vertebrate classes. The most commonly recorded haemogregarines to parasitize anurans are species of *Hepatozoon* Miller, 1908. To date 16 *Hepatozoon* species have been described from anurans in Africa, with only a single species, *Hepatozoon hyperolli* (Hoare, 1932), infecting a member of the Hyperoliidae. Furthermore, only two *Hepatozoon* species are known from South African anurans, namely *Hepatozoon theileri* (Laveran, 1905) and *Hepatozoon ixoxo* Netherlands, Cook and Smit, 2014, from *Amietia delalandii* (syn. *Amietia quecketti*) and three *Sclerophrys* species, respectively. Blood samples were collected from a total of 225 individuals representing nine hyperoliid species from several localities throughout northern KwaZulu-Natal, South Africa. Twenty frogs from three species were found positive for haemogregarines, namely *Afrixalus fornasinii* (6/14), *Hyperolius argus* (2/39), and *Hyperolius marmoratus* (12/74). Based on morphological characteristics, morphometrics and molecular findings three new haemogregarine species, *Hepatozoon involucrum* Netherlands, Cook and Smit n. sp., *Hepatozoon tenuis* Netherlands, Cook and Smit n. sp. and *Hepatozoon thori* Netherlands, Cook and Smit n. sp., are described from hyperoliid hosts. Furthermore, molecular analyses show anuran *Hepatozoon* species to be a separate monophyletic group, with species isolated from African hosts forming a monophyletic clade within this cluster.

## Introduction

Haemogregarines (Apicomplexa: Adeleiorina) are heteroxenous, intraerythrocytic or intraleucocytic parasites, infecting a broad range of vertebrate intermediate hosts including amphibians, reptiles, fishes, birds and mammals. These parasites are possibly transmitted by an equal diversity of haematophagous invertebrate definitive hosts or vectors, such as dipteran insects, ticks, mites, leeches and even gnathiid isopods (see Smith, 1996; Davies and Johnston, 2000; Curtis *et al.* 2013). Haemogregarines are currently divided into four families (Barta *et al.* 2012), namely Dactylosomatidae Jakowska and Nigrelli, 1955, Haemogregarinidae Léger, 1911, Hepatozoidae Miller, 1908, and Karyolysidae Labbé, 1894.

Within the Hepatozoidae, *Hepatozoon* Miller, 1908 is characterized by the presence of gamonts in erythrocytes or leucocytes, with no merogonic division occurring in the peripheral blood of the vertebrate host. Furthermore, *Hepatozoon* species are characterized by the pairing (syzygy) of gamonts in the definitive invertebrate host or vector following a blood meal. These paired gamonts then penetrate the gut wall and enter the haemocoel where sporogonic development and ultimately the formation of large oocysts occur. These thick-walled oocysts (also known as large multisporocystic oocysts) contain sporocysts with sporozoites, the infective stages of the parasite, which emerge upon the ingestion by the intermediate vertebrate host and give rise to merogonic stages in the liver (Desser *et al*. 1995; Smith, 1996; Barta, 2000).

*Hepatozoon* species are the most commonly reported haemogregarines to parasitize anurans. Currently, there are 45 recognized species from anurans globally, with 16 of these described from African hosts (see Smith, 1996; Netherlands *et al.* 2014*a*, *b*). According to Netherlands *et al*. (2014*a*), the majority of these species (12/16) were described from the Bufonidae, namely *Hepatozoon aegyptia* (Mohammed and Mansour, 1963), *Hepatozoon assiuticus* (Abdel-Rahman, El-Naffar, Sakla and Khalifa, 1978), *Hepatozoon boueti* (França, 1925), *Hepatozoon faiyumensis* (Mansour and Mohammed, 1966), *Hepatozoon francai* (Abdel-Rahman, El-Naffar, Sakla and Khalifa, 1978), *Hepatozoon froilanoi* (França, 1925), *Hepatozoon ixoxo* Netherlands, Cook and Smit, 2014, *Hepatozoon lavieri* (Tuzet and Grjebine, 1957), *Hepatozoon magni* (Hassan, 1992), *Hepatozoon moloensis* (Hoare, 1920), *Hepatozoon pestanae* (França, 1910), and *Hepatozoon tunisiensis* (Nicolle, 1904). Two species were described from the Ptychadenidae, namely *Hepatozoon epuluensis* (Van den Berghe, 1942), and *Hepatozoon neireti* (Laveran, 1905), and only a single species from the Pyxicephalidae and Hyperoliidae, namely *Hepatozoon theileri* (Laveran, 1905), and *Hepatozoon hyperolli* (Hoare, 1932), respectively. Apart from *Hepatozoon hyperolli,* which was described from an unidentified *Hyperolius* species in Uganda (Hoare, 1932), the only other *Hepatozoon* species reported from the Hyperoliidae are two unnamed species reported in *Hyperolius marmoratus* and *Hyperolius puncticulatus*, from northern KwaZulu-Natal (KZN), South Africa (Netherlands *et al.* 2015) and Amani, Tanzania (Ball, 1967), respectively. In South Africa, only two *Hepatozoon* species are known from anurans, namely *H. theileri* and *H. ixoxo*, from the pyxicephalid *Amietia delalandii* (syn. *Amietia quecketti*) and three *Sclerophrys* species (Bufonidae) respectively, namely *Sclerophrys pusilla* (syn. *Amietophrynus maculatus*), *Sclerophrys* (syn. *Amietophrynus*) *garmani* and *Sclerophrys* (syn. *Amietophrynus*) *gutturalis*.

Over the past decade several phylogenetic studies on adeleorinid parasites, using 18S rDNA sequences, have provided useful insight into the evolutionary relationships of this group, as well as the better capability to distinguish between species. However, because the 18S rRNA nuclear gene is a relatively conserved marker, it shows certain nodes to be unresolved (Barta *et al*. 2012; Maia *et al.* 2012; Haklová-Kočíková *et al.* 2014; Cook *et al.* 2016). In an effort to resolve these polytomies, a new genus *Bartazoon* Karadjian, Chavatte and Landau 2015, was proposed for species previously regarded as belonging to *Hepatozoon* parasitizing reptiles, amphibians, marsupials, birds and rodents, and was proposed to be transmitted solely by biting insects (Karadjian *et al.* 2015). However, the suggested life history of certain species within the proposed genus such as *Hepatozoon fitzsimonsi* (Dias, 1953) do not conform to the recommended characteristic defining *Bartazoon* (see Cook *et al.* 2014; Karadjian *et al.* 2015). Also as pointed out by Maia *et al.* (^30,31^), it is possible that *Hepatozoon perniciosum* Miller, 1908, the type species of the genus *Hepatozoon*, may, in fact, form part of the newly proposed genus *Bartazoon*, as most other rodent haemogregarine species do. Furthermore, increased work on the phylogenetic relationships of the haemogregarines continues to identify new genetic lineages, showing that *Bartazoon* is not a well-supported monophyletic group (Tomé *et al.* 2016; Maia *et al.* 2016*a*). Thus, to revise the deeper taxonomy (family and genus level) of haemogregarines based on their phylogenetic affinities and life histories, more studies using faster-evolving markers such as mitochondrial genes (e.g. Leveille *et al.* 2014), elucidating life cycles, and building larger datasets are necessary. Therefore, as suggested and used by Maia *et al.* (2016*b*) we will continue to refer to species parasitizing anuran hosts as species of *Hepatozoon* and not *Bartazoon*.

Prior to the study of Netherlands *et al.* (2014*a*) all the African anuran *Hepatozoon* species descriptions, ranging from the early 1900s till the late 1970s, were solely based on the morphology of the peripheral blood gamont stages. Unfortunately, many of these descriptions were scantily illustrated and incomplete, with almost 60% of the species described from the same host (*Sclerophrys regularis*) and in more or less the same geographical area (see Netherlands *et al.* 2014*a*, *b*). Thus many of these species may later need to be synonymized once more advanced and standardized methods are used to characterize these haemogregarines. In South Africa only five studies on amphibian haemogregarines have been carried out (Laveran, 1905; Fantham *et al*. 1942; Netherlands *et al.* 2014*a*, *b*; Netherlands *et al.* 2015). From these, only a single study was a multispecies haemoparasite survey across different anuran families (Netherlands *et al.* 2015), and although in that study several different haemogregarines were observed in anurans, only one hyperoliid species, *Hyp. marmoratus* (as mentioned above) contained a *Hepatozoon* species, which was not identified to species level.

Thus the objectives of the current study were (1) to establish which hyperoliid frog species in northern KZN, South Africa, contain haemogregarines. (2) to determine the species diversity of the haemogregarine parasites observed. (3) to ascertain if any of the haemogregarines found were previously described or reported species and (4) to compare any parasites characterized in the current study with available molecular data for anuran haemogregarines in order to determine their phylogenetic relationships.

## Materials and methods

### Frog collection and study area

**Fig. 1.**
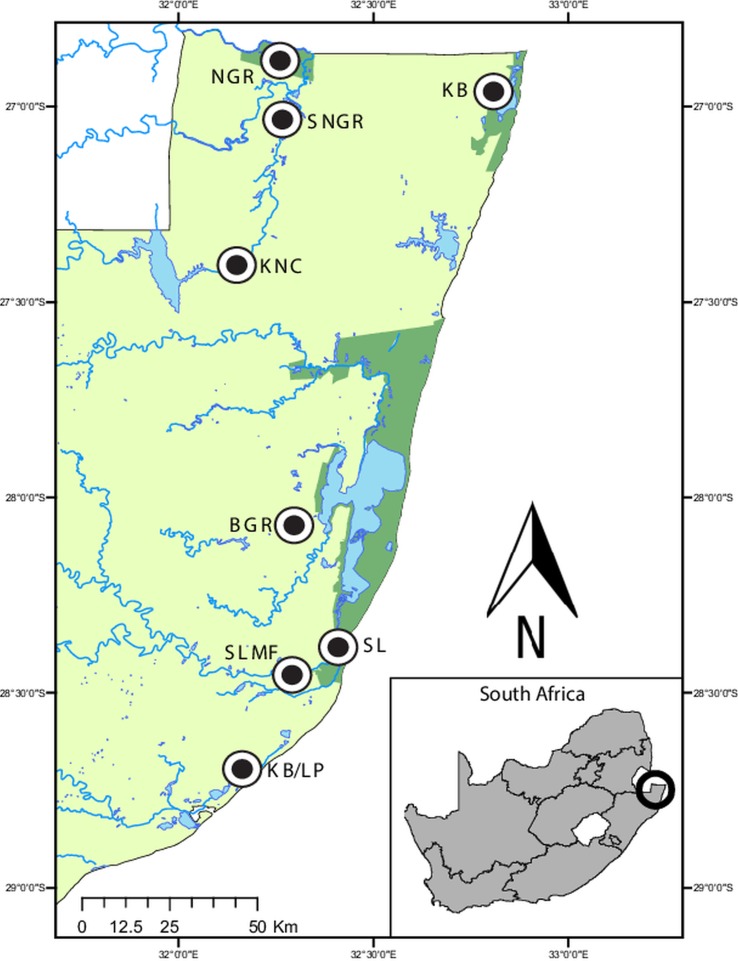
Map of the sampling localities in northern KwaZulu-Natal, South Africa. Ndumo Game Reserve (NGR) 26°52′00″S, 32°15′00″E, the area directly surrounding the NGR (SNGR) 27°00′13″S, 32°16′50″E, Kwa Nyamazane Conservancy (KNC) 27°23′35″S, 32°08′41″E, Bonamanzi Game Reserve (BGR) 28°03′25″S 32°17′42″E, Kosi Bay (KB) 26°57′16″S 32°48′07″E, KwaMbonambi/Langepan (KB/LP) 28°39′43″S 32°10′06″E, St. Lucia (SL) 28°23′10″S 32°24′29″E and St. Lucia Monzi Farm (SLMF) 28°26′56″S 32°17′18″E.

A total of 225 individuals representing nine hyperoliid species, were collected from several localities throughout northern KwaZulu-Natal, South Africa (Fig. 1), following the collection methods described in Netherlands *et al.* (2015). Frogs were identified using Du Preez and Carruthers (2009), and identifications were confirmed by one of the authors of this guide (LdP). After processing, all specimens were released at the site of capture. This study received the relevant ethical approval from the North-West University's AnimCare ethics committee (ethics number: NWU-00372-16-A5).

### Processing of samples and light microscopy screening

Blood (>0·1 mL) was taken from each frog *via* cardiac or femoral venipuncture and thin blood smears prepared on clean glass slides, air-dried, fixed and stained using Giemsa-stain (FLUKA, Sigma-Aldrich, Steinheim, Germany). The remaining blood was preserved in 70% ethanol for molecular work (ratio 1 : 15). Stained blood smears were screened at 1000× and images captured and measured using the imaging software NIS Elements Ver. 4 as described by Netherlands *et al.* (2015). Fifty mature gamonts were measured per *Hepatozoon* species. Measurements comprised the parasite's length (including recurved tail when present) and width within its parasitophorous vacuole (PV), and the parasite's nucleus length and width. Measurements of the PV length and width, and the length from mid nucleus to both anterior and posterior end of the parasite were also taken. Parasitaemia was calculated per 100 erythrocytes, with ~10^4^ erythrocytes examined per blood smear, following previous methods (see Cook *et al.* 2015*a*).

### DNA extraction, PCR amplification and phylogenetic analyses

Ethanol-preserved blood samples from parasitized frog specimens (*n* = 10) were used for molecular work. Two additional blood samples of *A. delalandii* parasitized with *H. theileri* and *S. pusilla* parasitized with *H. ixoxo* from a previous study (Netherlands *et al.* 2014*a*) were added to obtain longer comparative sequences as compared with the previous study by Netherlands *et al.* (2014*a*). Genomic DNA of haemogregarine species were extracted from the blood samples using the KAPA Express Extract Kit (Kapa Biosystems, Cape Town, South Africa). Once extracted, DNA was used for polymerase chain reaction (PCR) amplification. The PCR reactions targeted two fragments of approximately 940 and 1400 nt of the 18S rRNA gene. The 18S rRNA gene sequences were amplified using a combination of two primer sets based on previous studies of haemogregarines belonging to *Karyolysus* Labbé, 1894, *Hemolivia* Petit, Landau, Baccam and Lainson, 1990 and *Hepatozoon* (see Ujvari *et al.* 2004; Criado-Fornelio *et al.* 2006; Cook *et al.* 2015*b*, 2016). The first fragment was amplified using HAM-F (5′-GCCAGTAGTCATATGCTTGTC-3′) and HepR900 (5′-CAAATCTAAGAATTTCACCTCTGAC-3′) (see Ujvari *et al.* 2004; Criado-Fornelio *et al.* 2006), and the second fragment HepF300 (5′-GTTTCTGACCTATCAGCTTTCGACG-3′) and 2868 (5′-TGATCCTTCTGCAGGTTCACCTAC-3′) (see Medlin *et al.* 1988; Ujvari *et al.* 2004). Conditions for PCR were as follows: initial denaturation at 95 °C for 3 min, followed by 35 cycles, entailing a 95 °C denaturation for 30 s, annealing at 61 °C for 30 s with an end extension at 72 °C for 2 min, and following the cycles a final extension of 72 °C for 10 min. PCR reactions were performed with volumes of 25 *µ*L, using 12·5 *µ*L Thermo Scientific DreamTaq PCR master mix (2×) (final concentration: 2× DreamTaq buffer, 0·4 mm of each dNTP, and 4 mm MgCl_2_), 1·25 *µ*L (10 *µ*m) of each of the primer sets mentioned above, and at least 25 ng DNA. The final reaction volume was made up with PCR-grade nuclease-free water (Thermo Scientific). Reactions were undertaken in a Bio-Rad C1000 Touch™ Thermal Cycler PCR machine (Bio-Rad, Hemel Hempstead, UK). Resulting amplicons were visualized under ultraviolet light on a 1% agarose gel stained with gel red using a Bio-Rad GelDoc™ XR+ imaging system (Bio-Rad, Hemel Hempstead, UK). PCR products from each sample were sent to a commercial sequencing company (Inqaba Biotechnical Industries (Pty) Ltd, Pretoria, South Africa) for purification and sequencing in both directions. Resultant sequences were assembled, and chromatogram-based contigs were generated and trimmed using Geneious R9·1 (http://www.geneious.com, Kearse *et al.* 2012). Sequence and species identity was verified against previously published sequences using the Basic Local Alignment Search Tool (BLAST) (Altschul *et al.* 1990). Sequences obtained in the current study were deposited in the NCBI GenBank database under the following accession numbers [GenBank: MG041591–MG041605].

For comparison, all 18S rDNA sequences of anuran haemogregarines, longer than 1500 nt (comprising species of *Hepatozoon*, *Hemolivia, Babesiosoma* and *Dactylosoma*) as well as *Hepatozoon sipedon* Smith, Desser and Martin, 1994, [GenBank: JN181157] from the snake *Nerodia sipedon sipedon*, were downloaded from GenBank and aligned to the sequences generated in the current study. *Hepatozoon sipedon* was selected as it was shown by Barta *et al.* (2012) to be sister to *Hepatozoon catesbianae* (Stebbins, 1904) and *Hepatozoon clamatae* (Stebbins, 1905), at that point the only two species of *Hepatozoon* of frogs for which 18S rDNA sequences were available. Furthermore, *H. sipedon* first makes use of a frog intermediate host in which tissue development occurs before transmission to its second intermediate snake host (see Smith *et al.* 1994). Thus all species included in the analysis have an anuran host in their life cycle. Although there are other sequences available from a *Hepatozoon* species characterized from the anurans *Pelophylax perezi* [GenBank: KF733812] and *Leptodactylus chaquensis* [GenBank: JX987775], from the Azores in the North Atlantic Ocean, and Pantanal, Brazil respectively, they were not added to our analysis because these concerned shorter fragments (see Harris *et al.* 2013; Leal *et al.* 2015). *Babesiosoma stableri* Schmittner and McGhee, 1961 [GenBank: HQ224961] and *Dactylosoma ranarum* Lankester, 1871 [GenBank: HQ224957; HQ224958] were chosen as the outgroup, as they were shown by Barta *et al.* (2012) to belong to a sister group to our current ingroup. Sequences were aligned using the MUSCLE alignment tool (Edgar, 2004) under the default settings and implemented in Geneious R9·1. The alignment consisted of 14 sequences with a 1497 nt conserved region selected using the Gblocks 0·91b server (Castresana, 2000). To infer phylogenetic relationships both Bayesian inference (BI) and Maximum likelihood (ML) methods were used. The BI analysis was performed using MrBayes 3·2·2 (Huelsenbeck and Ronquist, 2001) and the ML analysis was performed using RAxML Ver. 7·2·8. (Stamatakis, 2014) both implemented from within Geneious R9·1. Prior to the analyses, a model test was performed to determine the most suitable nucleotide substitution model, according to the Akaike information criterion using jModelTest 2·1·7 (Guindon and Gascuel, 2003; Darriba *et al.* 2012). The model with the best AICc score was the Transitional model (Posada, 2003) with estimates of invariable sites and a discrete Gamma distribution (TVM + I + Γ). However, this model was substituted by the General Time Reversible (Tavaré, 1986) model (GTR + I + Γ) in MrBayes and in RAxML, as this was the next model available with the best AICc score. For the BI analysis, the Markov Chain Monte Carlo (MCMC) algorithm was run for 10 million generations, sampling every 100 generations, and using the default parameters. The first 25% of the trees were discarded as ‘burn-in’ with no ‘burn-in’ samples being retained. Results were visualized in Trace (implemented from within Geneious R9·1), to assess convergence and the burn-in period. For the ML analysis, nodal support was assessed using 1000 rapid bootstrap inferences. Model-corrected (TVM + I + Γ) genetic distances were calculated in PAUP version 4.0a152 (Swofford, 2002), with the assumed proportion of invariable sites = 0·598 and the gamma shape parameter = 0·775.

## Results

**Fig. 2.**
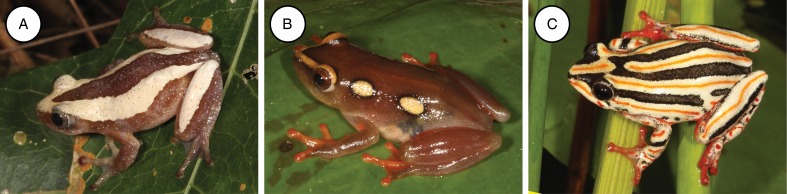
Three frog species found positive for haemogregarines. (A) *Afrixalus fornasinii*, (B) *Hyperolius argus*, and (C) *Hyperolius marmoratus*.

A total of 225 individuals representing nine species from the family Hyperoliidae, namely *Afrixalus aureus* (*n* = 18), *Afrixalus delicatus* (*n* = 13), *Afrixalus fornasinii* (*n* = 14), *Hyperolius argus* (*n* = 39), *Hyperolius marmoratus* (*n* = 74), *Hyperolius tuberlinguis* (*n* = 38), *Hyperolius pusillus* (*n* = 14), *Kassina senegalensis* (*n* = 9), and *Phylctimantis* (syn. *Kassina*) *maculatus* (*n* = 6) were collected and screened for haemogregarines. Twenty frogs (8·9%) from three species were found positive for haemogregarines, specifically *A. fornasinii* (6/14), *Hyp. argus* (2/39), and *Hyp. marmoratus* (12/74) (see Fig. 2A–C). Based on peripheral blood stages, the haemogregarines of the current study conform to the genus *Hepatozoon*. Although possible meront stages were observed in the peripheral blood for one species, these were rare and no merogonic division was detected. Furthermore, these haemogregarines did not compare with the closely related genus *Hemolivia*, as no schizogony or cyst formation in the erythrocytes of the hosts was observed.

### Species descriptions

Phylum: Apicomplexa Levine, 1970
Class: Conoidasida Levine, 1988
Order: Eucoccidiorida Léger & Duboscq, 1910
Suborder: Adeleorina Léger, 1911
Family: Hepatozoidae Wenyon, 1926
Genus: *Hepatozoon* Miller, 1908

*Hepatozoon involucrum* Netherlands, Cook and Smit n. sp.

*Type-host: Hyperolius marmoratus* Rapp, 1842 (Anura: Hyperoliidae).

*Vector:* Unknown.

*Type-locality:* The specimens were collected in the Kwa Nyamazane Conservancy (KNC), KwaZulu-Natal, South Africa (27°23′35″S, 32°08′41″E).

*Other localities:* St. Lucia on Monzi Farm, KwaZulu-Natal, South Africa (28°26′56″S 32°17′18″E).

*Type-material:* Hapantotype, 1× blood smear from *Hyp. marmoratus* deposited in the protozoan collection of the National Museum, Bloemfontein, South Africa under accession number NMB P 467; parahapantotype, 1× blood smear from *Hyp. marmoratus*; deposited in the Protozoan Collection of the National Museum, Bloemfontein (NMB), South Africa, under accession number NMB P 468.

*Representative DNA sequences:* The 18S rRNA gene sequences have been submitted to the GenBank database under the accession numbers MG041591–MG041594.

*ZooBank registration:* The Life Science Identifier (LSID) of the article is urn:lsid:zoobank.org:pub:F73407D7-1E08-4C3C-B066-889058B77C4C. The LSID for the new name *Hepatozoon involucrum* Netherlands, Cook and Smit is urn:lsid:zoobank.org:act:A43D46E8-5C9F-4405-8907-94D7B02EAEA7.

*Etymology:* The species epithet is derived from the Latin word *involucrum* meaning envelope or sheath, and is based on the prominent PV encircling the gamont.

**Fig. 3.**
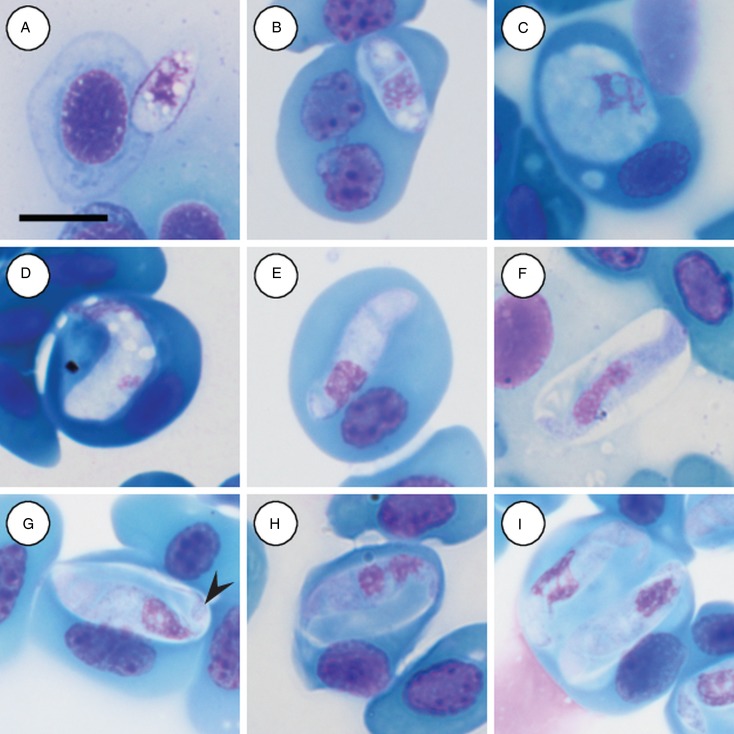
*Hepatozoon involucrum* n. sp. in the reed frog *Hyperolius marmoratus*. (A and B) Trophozoite. (C) Possible meront stage. (D) Possible vacuolated meront stage. (E) Immature gamont stage. (F) Extracellular or free gamont. (G, arrowhead) Mature gamont displaying a recurved tail. (H) Mature gamont, note the expanding parasite nucleus and large parasitophorous vacuole. (I) Double infection of a single erythrocyte. All images captured from the deposited slides (NMB P 467 & 468). Scale bar: 10 *µ*m.

Description:

Trophozoites: rare, occurring singularly within erythrocytes, oval to rounded, measuring 12·2–12·5 (12·3 ± 0·2) *μ*m long × 4·8–5·7 (4·2 ± 0·6) *μ*m wide (*n* = 2) with finely vacuolated cytoplasm staining whitish-pink (Fig. 3A and B), note lysis of the host cell nucleus (Fig. 3B). Nucleus containing loosely arranged chromatin, staining pink, measuring 3·7–5·2 (4·5 ± 1·0) *μ*m long × 3·2–4·9 (4·0 ± 1·2) *μ*m wide (*n* = 2). Mid nucleus position measuring 5·8–7·4 (6·6 ± 1·2) *μ*m to anterior, and 5·4–5·6 (5·5 ± 0·1) *μ*m to posterior.

Meronts: rare, irregular in shape, often with a foamy cytoplasm, staining whitish-blue to purple (Fig. 3C and D), and measuring 9·5 *µ*m long × 8·8 *µ*m wide (*n* = 1). Nucleus containing loosely arranged chromatin, staining pink to purple, measuring 6·8 *µ*m long  × 3·7 *µ*m wide (*n* = 1).

Immature gamonts: elongated with small-recurved tail, within a vaguely visible PV, cytoplasm staining whitish-purple, causing displacement of the host cell nucleus (Fig. 3E). Parasite (including recurved tail) measuring 16·4–23·0 (19·8 ± 1·8) *μ*m long × 4·4–5·7 (5·1 ± 0·4) *μ*m wide (*n* = 10), PV measuring 14·2–18·4 (15·6 ± 1·3) *μ*m long × 5·2–9·1 (6·5 ± 1·5) *μ*m wide (*n* = 10). Nucleus rounded, usually situated in the posterior half of the parasite, loosely arranged chromatin, staining purple, and measuring 3·0–7·0 (5·4 ± 1·4) *μ*m long × 2·6–5·6 (3·8 ± 0·9) *μ*m wide (*n* = 10). Mid nucleus position measuring 10·0–13·7 (11·7 ± 1·4) *μ*m to anterior side, and 6·6–11·1 (8·6 ± 1·6) *μ*m to posterior side (*n* = 10).

Mature gamonts: elongated and oval, encased in a large PV (Fig. 3F–I); often recurved at both the anterior and posterior poles, and in some cases a clear recurved tail is visible (Fig. 3G arrowhead); infrequent extracellular or free moving gamont (Fig. 3F), as well as single erythrocytes parasitised by two gamonts (Fig. 3I); gamonts cause noticeable displacement of the host cell nucleus. Parasite (including recurved tail) measuring 18·7–25·9 (21·8 ± 1·5) *μ*m long × 4·0–6·3 (5·1 ± 0·5) *μ*m wide (*n* = 50), PV measuring 16·5–20·9 (18·3 ± 1·0) *μ*m long × 6·3–10·8 (8·3 ± 1·1) *μ*m wide (*n* = 50). Nucleus elongated or loosely arranged, usually situated in the posterior half of the parasite, loose chromatin strands often visible, staining purely-pink, and measuring 4·8–8·9 (6·4 ± 0·9) *μ*m long × 2·2–4·2 (3·2 ± 0·4) *μ*m wide (*n* = 50). Mid nucleus position measuring 8·4–19·9 (13·8 ± 1·8) *μ*m to anterior side, and 5·4–11·6 (8·2 ± 1·4) *μ*m to posterior side (*n* = 50). Parasitaemia of all infected individuals (*n* = 7) in percentage (%) was 1·0–30·0 (8·0 ± 2·0).

Remarks:

Based on the morphology and morphometrics of peripheral blood stages in *Hyp. marmoratus*, *H. involucrum* n. sp. does not conform morphologically to any of the 16 currently recognized *Hepatozoon* species in African anurans. The only other named species infecting a member of the Hyperoliidae, is *H. hyperolii*, and can be distinguished from *H. involucrum* n. sp. based on the shape of the former parasite's gamont. The gamont of *H. hyperolii* is cylindrical with rounded ends and a long recurved tail folded onto itself in the absence of a prominent PV (see Fig. 6A–C). In contrast the gamont of *H. involucrum* n. sp. has an elongated and encased gamont, which is often recurved at both the anterior and posterior poles. The mean length and width of *H. involucrum* n. sp., which includes the parasite's PV, is 18·3 *µ*m long × 8·3 *µ*m wide. Although these mean length measurements do overlap with several species namely, *H. faiyumensis*, *H. francai*, *H. moloensis* and *H. neireti*, the mean width in combination with the length of these species does not conform. Overall the gamont measurements of *H. involucrum* n. sp. compare closest to those of *H. moloensis* (18·8 *µ*m long × 7·8 *µ*m wide), which was described from an unidentified *Sclerophrys* species in Molo, Kenya (see Hoare, 1920). However, the oval shape, recurved tail and absence of a PV in *H. moloensis* are distinctive and distinguishable from *H. involucrum* n. sp. as described above. Similarly, these distinctive characteristics of *H. involucrum* n. sp, which differentiate it from *H. moloensis*, also differentiate it from other African anuran species of *Hepatozoon*.

In South Africa a *Hepatozoon* species corresponding morphologically to *H. involucrum* n. sp. was reported from the same host and area in an anuran biodiversity blood parasite survey by Netherlands *et al.* (2015), however, this parasite was not formally described or named (see Netherlands *et al.* 2015, Fig. 2D).

Globally the species that conforms most closely to *H. involucrum* n. sp. is *Hepatozoon nucleobisecans* (Shortt, 1917) described from the Indian toad *Duttaphrynus melanostictus* (syn. *Bufo melanostictus*). Although the reported gamont length (18·3 *µ*m long) of *H. nucleobisecans*, including the PV, equals the mean length of *H. involucrum* n. sp., the width (4·8 *µ*m wide) is almost half. Furthermore, the gamont of *H. nucleobisecans* is not recurved at both the anterior and posterior poles within the PV (see Shortt, 1917).

*Hepatozoon tenuis* Netherlands, Cook and Smit n. sp.

*Type-host: Afrixalus fornasinii* (Bianconi, 1849) (Anura: Hyperoliidae).

*Other hosts: Hyperolius argus* Peters, 1854; *Hyperolius marmoratus* (Anura: Hyperoliidae).

*Vector:* Unknown.

*Type-locality:* The specimens were collected in St. Lucia on Monzi Farm, KwaZulu-Natal, South Africa (28°26′56″S 32°17′18″E).

*Other localities:* KwaMbonambi/Langepan, KwaZulu-Natal, South Africa (28°39′43″S 32°10′06″E).

*Type-material:* Hapantotype, 1× blood smear from *A. fornasinii* deposited in the protozoan collection of the National Museum, Bloemfontein, South Africa under accession number NMB P 469; parahapantotypes, 1× blood smear from *A. fornasinii*, and *Hyperolius marmoratus*; deposited in the Protozoan Collection of the National Museum, Bloemfontein (NMB), South Africa, under accession numbers NMB P 470 and NMB P 471, respectively.

*Representative DNA sequences:* The 18S rRNA gene sequences have been submitted to the GenBank database under the accession numbers MG041595–MG041599.

*ZooBank registration:* The Life Science Identifier (LSID) of the article is urn:lsid:zoobank.org:pub:F73407D7-1E08-4C3C-B066-889058B77C4C. The LSID for the new name *Hepatozoon tenuis* Netherlands, Cook and Smit is urn:lsid:zoobank.org:act:AD607D8B-D43D-49C6-8139-2782306FE2F5.

*Etymology:* The species epithet is derived from the Latin word *tenuis*, which means thin or slender. This refers to the long slender shape of the gamont.

**Fig. 4.**
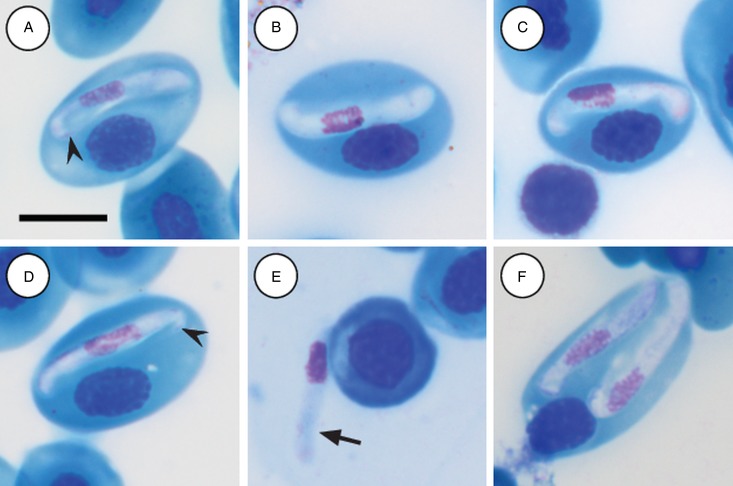
*Hepatozoon tenuis* n. sp. mature gamonts parasitizing erythrocytes in the folding leaf frog *Afrixalus fornasinii* (A–C) and the reed frogs *Hyperolius marmoratus* (D) and *Hyperolius argus* (E and F). (A–C) Close-fitting parasitophorous vacuole, visible on the concave side of the gamont. (A and D, arrowhead) Gamont with a recurved tail. (E, arrow) Extracellular or free gamont. (F) Double infection of a single erythrocyte. All images captured from the deposited slides (NMB P 469–471). Scale bar: 10 *µ*m.

Description:

Mature gamonts: slender and elongated, with a pinkish-white staining cytoplasm, within a close-fitting PV visible on the concave side of the gamont (Fig. 4A–C); in some cases a recurved tail is visible (Fig. 4A and D arrowhead); also an occasional extracellular or free moving gamont, (Fig. 3E arrow), as well as a single erythrocyte parasitised by two gamonts (Fig. 4F); gamonts cause obvious displacement of the host cell nucleus. Parasites (including recurved tail when visible) measuring 11·2–16·8 (13·9 ± 1·6) *μ*m long × 3·7– 6·7 (4·8 ± 0·6) *μ*m wide (*n* = 50), PV measuring 17·8–20·7 (19·4 ± 0·8) *μ*m long × 5·0–7·5 (6·7 ± 0·4) *μ*m wide (*n* = 50). Nucleus elongated and neatly arranged, usually situated in the posterior half of the parasite, loose chromatin staining purely-pink, and measuring 2·1–5·2 (3·9 ± 0·6) *μ*m long × 1·6–4·9 (10·8 ± 0·9) *μ*m wide (*n* = 50). Mid nucleus position measuring 4·8–9·4 (6·7 ± 1·1) *μ*m to anterior, and 4·6–10·1 (7·2 ± 1·2) *μ*m to posterior (*n* = 50). Parasitaemia of all infected individuals (*n* = 9) calculated in percentage (%) was 1·0–35·0 (6·0 ± 2·0), two (*Hyp. argus* and *Hyp. marmoratus*) of the nine infected individuals contained mixed infections the parasite described below.

Remarks:

*Hepatozoon tenuis* n. sp. parasitising *A. fornasinii*, *Hyp. argus* and, *Hyp. marmoratus*, can be distinguished from *H. involucrum* n. sp., based on the difference in gamont morphometrics. Morphologically, gamonts have an overall similar appearance to *H. involucrum* n. sp., however, gamonts of *H. involucrum* n. sp. measure a mean of 21·8 *µ*m long × 5·1 *µ*m wide (*n* = 50) (PV not included) and a mean of 18·3 *µ*m long × 8·3 *µ*m wide (*n* = 50) (PV included), as compared with gamonts of *H. tenuis* n. sp. measuring a mean of 13·9 *µ*m long × 4·8 *µ*m wide (*n* = 50) (PV not included) and a mean of 19·4 *µ*m long × 6·7 *µ*m wide (*n* = 50) (PV included). This slender looking parasite can be distinguished from other anuran *Hepatozoon* species based on the marginally visible PV, as well as often being recurved at both the anterior and posterior poles within the PV.

*Hepatozoon thori* Netherlands, Cook and Smit n. sp.

*Type-host: Hyperolius marmoratus* Rapp, 1842 (Anura: Hyperoliidae).

*Other hosts: Hyperolius argus*; *Hyperolius puncticulatus* (Pfeffer, 1893) (Anura: Hyperoliidae).

*Vector:* Unknown.

*Type-locality:* The specimens were collected in the Kwa Nyamazane Conservancy (KNC), KwaZulu-Natal, South Africa (27°23′35″S, 32°08′41″E).

*Other localities:* KwaMbonambi/Langepan, KwaZulu-Natal, South Africa (28°39′43″S 32°10′06″E); Amani, Tanzania.

*Type-material:* Hapantotype, 1 × blood smear from *Hyp. marmoratus* deposited in the protozoan collection of the National Museum, Bloemfontein, South Africa under accession number NMB P 472; parahapantotype, 1 × blood smear from *Hyp. marmoratus*; deposited in the Protozoan Collection of the National Museum, Bloemfontein, South Africa, under accession number NMB P 473.

*Representative DNA sequences:* The 18S rRNA gene sequences have been submitted to the GenBank database under the accession numbers MG041600–MG041603.

*ZooBank registration:* The Life Science Identifier (LSID) of the article is urn:lsid:zoobank.org:pub:F73407D7-1E08-4C3C-B066-889058B77C4C. The LSID for the new name *Hepatozoon thori* Netherlands, Cook and Smit is urn:lsid:zoobank.org:act:00CD84D9-D6A8-4B41-A048-DFD0DBF4B045.

*Etymology:* The species epithet is derived from Norse mythology after the hammer-wielding god Thor. This is based on the hammer-like shape of the gamont.

**Fig. 5.**
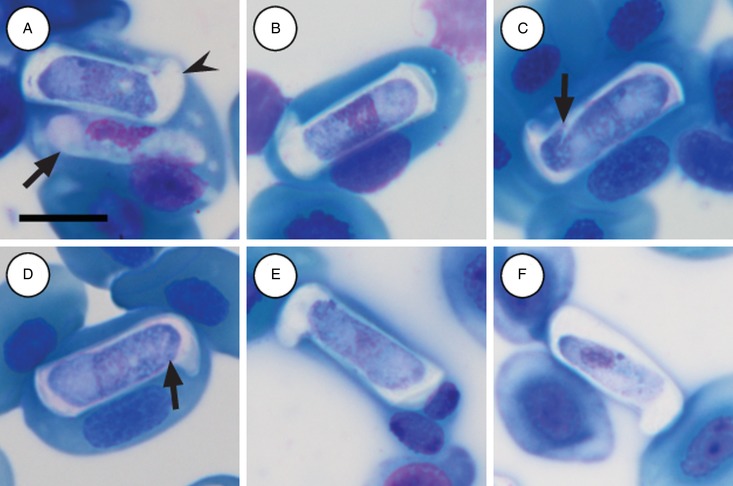
*Hepatozoon thori* n. sp. gamonts parasitizing erythrocytes in the reed frogs *Hyperolius marmoratus* (A–C) and *Hyperolius argus* (D–F). (A) Double infection of a single erythrocyte, with an immature (arrow) and mature (arrowhead) gamont. (B–F) Prominent hammer-like or boot-shaped parasitophorous vacuole, allowing only a certain portion of the gamont to be visible. (C and D, arrow) Gamont displaying a short recurved tail. (E) Gamont causing the host cell nucleus to lyse. (F) Extracellular or free gamont. All images captured from the deposited slides (NMB P 472 & 473). Scale bar: 10 *µ*m.

Description:

Immature gamonts: rare, elongated without a visible PV, cytoplasm staining whitish-purple, measured 18·7 *µ*m long by 5·5 *µ*m wide (*n* = 1), causing displacement of the host cell nucleus and found parasitizing a single erythrocyte together with a mature gamont (Fig. 5A arrow). Nucleus rounded, situated in the posterior half of the parasite, loosely arranged chromatin, staining purple, and measuring 8·1 *µ*m long × 2·7 *µ*m wide (*n* = 1). Mid nucleus position measured 8·9 *µ*m to anterior side, and 9·8 *µ*m to posterior side (*n* = 1).

Mature gamonts: elongated, causing displacement of the host cell nucleus. Encased in a prominent hammer-like or boot-shaped PV, with a pseudopodial-like projection (Fig. 5A–F); occasionally a short recurved tail is visible (Fig. 5C and D arrow); mature gamonts cause the host cell nucleus to lyse (Fig. 5E); extracellular or free moving gamont, possibly probing to enter new host cell (Fig. 5F). Parasite measuring 11·2–16·8 (13·9 ± 1·6) *μ*m long × 3·7– 6·7 (4·8 ± 0·6) *μ*m wide (*n* = 50), with the PV measuring 17·8–20·7 (19·4 ± 0·8) *μ*m long × 5·0–7·5 (6·7 ± 0·4) *μ*m wide (*n* = 50). Parasites, including the recurved tail (see Fig. 5C and D arrow), measuring 19·1–21·7 (20·4 ± 1·1) *μ*m long (*n* = 5). Nucleus elongated or loosely arranged, usually situated in the posterior half of the parasite, loose chromatin strands often visible, staining purely-pink, and measuring 2·1–5·2 (3·9 ± 0·6) *μ*m long × 1·6–4·9 (10·8 ± 0·9) *μ*m wide (*n* = 50). Mid nucleus position measured 4·8–9·4 (6·7 ± 1·1) *μ*m to anterior, and 4·6–10·1 (7·2 ± 1·2) *μ*m to posterior (*n* = 50). Parasitaemia of all infected individuals (*n* = 6) in percentage (%) was 1·0–21·0 (3·0 ± 2·0), two (*Hyp. argus* and *Hyp. marmoratus*) of the six infected individuals contained mixed infections with *H. tenuis* n. sp.

**Fig. 6.**
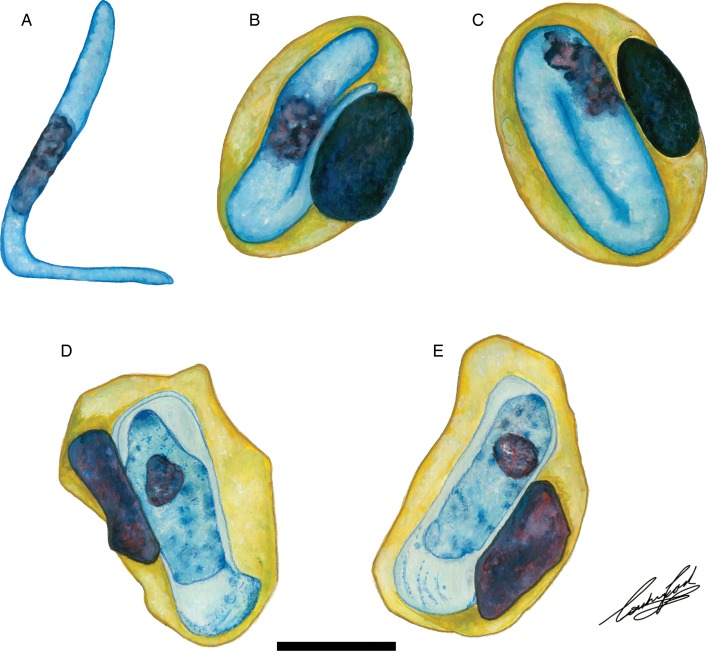
Illustrations of haemogregarine blood parasites in African hyperoliids. (A–C) *Hepatozoon hyperolii* (Hoare, 1932), described from an unidentified *Hyperolius* species in Uganda. Redrawn and adapted from Hoare (1932). (D and E) Unnamed *Hepatozoon* species reported in *Hyperolius puncticulatus*, from Amani, Tanzania. Redrawn and adapted from Ball (1967). Scale bar: 10 *µ*m.

Remarks:

*Hepatozoon thori* n. sp. parasitizing *Hyp. argus* and *Hyp. marmoratus* can be distinguished from *H. involucrum* n. sp., *H. tenuis* n. sp., and other anuran *Hepatozoon* species based on the distinctive shape of the hammer-like or boot-shaped PV that has a pseudopodial-like projection. The mean length and width of the parasite measure 13·9 *µ*m long × 4·8 *µ*m wide (PV not included) and 19·4 *µ*m long × 6·7 *µ*m wide (*n* = 50) (PV included). Based on the size and shape, the only other haemogregarine *H. thori* n. sp. conforms closest to is an unnamed *Hepatozoon* species (see Fig. 6D and E), measuring a mean of 14·1 *µ*m long × 4·8 *µ*m wide (PV not included) and 20·8 *µ*m long × 6·7 *µ*m wide (PV included). This unnamed species was reported in *Hyperolius puncticulatus*, from Amani, Tanzania (see Ball, 1967) (see below).

### Phylogenetic analysis

Amplicons of between 1640 and 1701 nt were derived from *H. involucrum* n. sp., *H. tenuis* n. sp., and *H. thori* n. sp. from the blood of *A. fornasinii, Hyp. argus* and *Hyp. marmoratus*. Additionally, sequences of *H. ixoxo* and *H. theileri*, were amplified from the blood collected in a previous study (Netherlands *et al.* 2014*a*) from *S. pusilla* and *A. delalandii,* respectively. The details of sequences used in the analyses are presented in Table 1.

**Table 1.** List of the sequence (18S rDNA) information used in the current study

Accession no.	Species	Host	Reference
MG041591	*Hepatozoon involucrum* n. sp.	*Hyperolius marmoratus*	Current study
MG041596	*Hepatozoon tenuis* n. sp.	*Afrixlus fornasinii*	Current study
MG041598	*Hepatozoon tenuis* n. sp.	*Hyperolius argus*	Current study
MG041599	*Hepatozoon tenuis* n. sp.	*Hyperolius marmoratus*	Current study
MG041600	*Hepatozoon thori* n. sp.	*Hyperolius argus*	Current study
MG041601	*Hepatozoon thori* n. sp.	*Hyperolius marmoratus*	Current study
MG041605	*Hepatozoon theileri*	*Ametia delalandii*	Current study
MG041604	*Hepatozoon ixoxo*	*Sclerophrys pusilla*	Current study
HQ224962	*Hepatozoon* cf. *clamatae*	*Rana clamitans*	Barta *et al.* (2012)
HQ224963	*Hepatozoon* cf. *clamatae*	*Rana clamitans*	Barta *et al.* (2012)
HQ224954	*Hepatozoon* cf. *catesbianae*	*Rana catesbeiana*	Barta *et al.* (2012)
HQ224960	*Hepatozoon magna*	*Pelophylax* kl. *esculentus*	Barta *et al.* (2012)
JN181157	*Hepatozoon sipedon*	*Nerodia sipedon sipedon*	Barta *et al.* (2012)
KP881349	*Hemolivia stellata*	*Amblyomma rotondatum* ex *Rhinella marina*	Karadjian *et al.* (2015)
HQ224961	*Babesiosoma stableri*	*Rana septentrionalis*	Barta *et al.* (2012)
HQ224957	*Dactylosoma ranarum*	*Pelophylax* kl. *esculentus*	Barta *et al.* (2012)
HQ224958	*Dactylosoma ranarum*	*Pelophylax* kl. *esculentus*	Barta *et al.* (2012)

The table includes the GenBank accession number, species, host species and the reference study.

Based on 1497 nt sequence comparisons of the 18S rRNA gene (see Table 2), the interspecific divergence (model-corrected genetic distance) between *H. involucrum* n. sp. and its closest relative *H. tenuis* n. sp. was 1·0%. *Hepatozoon involucrum* n. sp. and *H. thori* n. sp. had an interspecific divergence of 2·0%, and *H. tenuis* n. sp. and *H. thori* n. sp. differed by 1·8%. The interspecific divergence between the *Hepatozoon* species parasitizing anuran hosts and *Hepatozoon sipedon* [GenBank: JN181157] was between 7·7 and 10·6%. The intergeneric divergence between the *Hepatozoon* species parasitising anuran hosts, and *Hemolivia stellata* Petit, Landau, Baccam and Lainson, 1989 [GenBank: KP881349], *B. stableri* [GenBank: HQ224961] and *D. ranarum* [GenBank: HQ224957; HQ224958] were between 4·9–5·8%, 8·8–9·6% and 8·5–9·7%, respectively (Table 2).

**Table 2.** Estimates of divergence between partial 18S rDNA sequences from the haemogregarine species used in the current study

Species	1	2	3	4	5	6	7	8	9	10	11	12	13	14	15
1. MG041591 *H. involucrum* n. sp.															
2. MG041596 *H. tenuis* n. sp.	1·0														
3. MG041598 *H. tenuis* n. sp	1·0	0·2													
4. MG041601 *H. thori* n. sp.	2·0	1·8	1·9												
5. MG041605 *H. theileri*	2·1	1·8	1·9	2·1											
6. MG041604 *H. ixoxo*	1·7	1·4	1·4	1·5	1·4										
7. HQ224962 *H.* cf. *clamatae*	2·5	2·2	2·3	2·2	2·4	1·6									
8. HQ224963 *H.* cf. *clamatae*	2·6	2·4	2·5	2·4	2·6	1·7	0·1								
9. HQ224954 *H.* cf. *catesbianae*	2·5	2·2	2·3	2·2	2·4	1·6	0·1	0·3							
10. HQ224960 *H. magna*	2·2	1·7	1·8	1·9	2·1	1·6	1·8	2·0	1·8						
11. JN181157 *H. sipedon*	10·1	9·9	10·0	10·4	10·1	9·6	10·2	10·5	10·2	9·3					
12. KP881349 *Hemolivia stellata*	5·7	5·3	5·2	5·6	5·2	5·5	5·6	5·8	5·6	4·9	7·7				
13. HQ224961 *B. stableri*	9·0	9·0	9·2	9·4	8·9	9·6	9·1	9·3	9·3	9·0	10·6	5·0			
14. HQ224957 *D. ranarum*	9·1	9·1	9·3	9·5	9·0	9·7	9·2	9·4	9·4	9·1	10·7	5·1	0·6		
15. HQ224958 *D. ranarum*	8·7	8·6	8·8	9·0	8·5	9·3	8·8	9·0	9·0	8·6	10·3	4·7	0·3	0·0	

Distance matrix showing ranges for the model-corrected genetic distances between the sequences. Alignment length 1497 nt. Genetic distances shown as percentage (%).

**Fig. 7.**
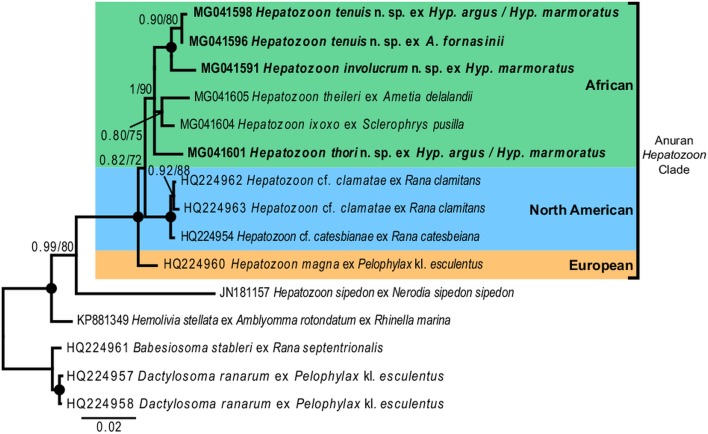
Consensus phylogram of anuran haemogregarines based on 18S rDNA sequences. Tree topologies for both Bayesian inference (BI) and Maximum Likelihood (ML) analyses were similar (represented on the BI tree), showing the phylogenetic relationships for *H. involucrum* n. sp., *H. tenuis* n. sp., and *H. thori* n. sp. (represented in bold), compared to other species of anuran *Hepatozoon* (with the exception of *Hepatozoon sipedon*), *Hemolivia*, and three species from the Dactylosomatidae as outgroup. Clades that neither produced 0·80 posterior probability (BI) or 70 bootstrap (ML) nodal support values were collapsed. Black circles represent 100% support for both BI/ML. The scale bar represents 0·02 nucleotide substitutions per site.

For the phylogenetic analyses the topologies of both the BI and ML trees were similar. The analyses showed *Hemolivia stellata* [GenBank: KP881349] as a well-supported sister taxon to the *Hepatozoon* species cluster, with *H. sipedon* [GenBank: JN181157] shown to be a sister species to a well-supported monophyletic clade comprising *Hepatozoon* species isolated from anuran hosts. The *Hepatozoon* species isolated from African and North American anurans formed two well-supported monophyletic clades, respectively, and were separate from the European species *H. magna* [GenBank: HQ224960]. The African *Hepatozoon* clade represents a polytomy with *H. involucrum* n. sp. and *H. tenuis* n. sp., forming a well-supported monophyletic clade and *H. ixoxo* and *H. theileri*, forming a poorly-supported clade, nested within this polytomy and separate to *H. thori* n. sp. (Fig. 7).

## Discussion

In the present study, we screened the peripheral blood of 225 individual frogs from nine species within the Hyperoliidae. Six species (*A. aureus*, *A. delicates*, *Hyp. tuberlinguis*, *Hyp. pusillus*, *K. senegalensis* and *P. maculatus*), totalling 205 specimens were found negative for haemogregarine parasites. Only 20 frogs from three species were found positive, namely *A. fornasinii* (6/14), *Hyp. argus* (2/39), and *Hyp. marmoratus* (12/74).

Morphological and molecular data indicate that the haemogregarines parasitising these hosts represent three distinct species of *Hepatozoon*, herein described as *H. involucrum* n. sp. parasitising *Hyp. marmoratus*; *H. tenuis* n. sp., parasitising *A. fornasinii*, *Hyp. argus* and *Hyp. marmoratus*; and *H. thori* n. sp. parasitising *Hyp. argus* and *Hyp. marmoratus*. Mature gamonts of *H. involucrum* n. sp. are characterized by the prominent PV encircling the large gamont, as well as the recurved ends of both poles of the gamont. When compared with *H. tenuis* n. sp., the overall appearance and characteristics are similar, except for a difference in size of the gamont and PV. The interspecific divergence between these two species is 1·0%. This has been shown in several studies to correspond to species-level differences in haemogregarines and for the slow evolving 18S rRNA marker (see Barta *et al.* 2012; Cook *et al.* 2015*b*; Borges-Nojosa *et al.* 2017). *Hepatozoon thori* n. sp. can be distinguished from both *H. involucrum* n. sp. and *H. tenuis* n. sp. based on the distinctive hammer-like shape of the gamont's PV. The interspecific divergence between *H. thori* n. sp., *H. involucrum* n. sp. and *H. tenuis* n. sp. was 2·0% and 1·8%, respectively.

The only other named species of *Hepatozoon* infecting a member of the Hyperoliidae is *H. hyperolii* described in an unidentified *Hyperolius* species by Hoare (1932), this parasite being vermicular in shape and folding over on itself within its host erythrocyte (see Fig. 6A–C) and therefore does not conform to any of the *Hepatozoon* species of the present study. However, Ball (1967) reported a second, but unnamed species in *Hyperolius puncticulatus* from Amani, Tanzania, and this species conforms both in size and shape to *H. thori* n. sp. (see Fig. 6D and E). In the current study, we propose that these two species are the same, despite parasitizing different hosts and possibly being geographically isolated. However, to confirm this, molecular data for this species from Amani, Tanzania is required.

In our phylogenetic analysis, *Hepatozoon* species isolated from anuran hosts formed a well-supported monophyly, separate to other closely related species of *Hepatozoon*. Furthermore, the African clade formed a monophyly, with *H. thori* n. sp. separate from the other species within this clade. *Hepatozoon involucrum* n. sp. and *H. tenuis* n. sp. form a well-supported monophyletic clade nested within the larger African clade. With an interspecific divergence of 1·0% (model-corrected distance), these two species are closely related, which concurs with their close morphological resemblance. *Hepatozoon ixoxo* and *H. theileri* form a less well supported (0·80/75) monophyletic group. The BI statistical information for the bipartitions of this group showed that apart from the 80% probability support, only 13% included *H. thori* n. sp. as part of this clade and 9% showed *H. theileri* formed a clade with *H. involucrum* n. sp. and *H. tenuis* n. sp., thus explaining the low support of this group. Furthermore, *H. ixoxo* and *H. theileri* differ considerably in morphological structure (see Conradie *et al.* 2017), and if compared with the phylogenetic and morphological relationship of *H. involucrum* n. sp. and *H. tenuis* n. sp. (as mentioned above), the former two species are not expected to be sister species. This underlines the importance of increased taxon sampling for these parasites, as the addition of more species to this dataset could result in better-supported clades and the polotomy of the African clade could be resolved. Additionally, faster-evolving markers (e.g. mtDNA) may further explain the biogeography and evolutionary history of these species globally. However, to date, only one haemogregarine, *H. catesbianae* isolated from the frog *Rana catesbeiana* has mtDNA sequence data available (see Leveille *et al.* 2014). Although these markers (mtDNA) may be complementary in providing an evolutionary perspective among these parasite groups, a lot more data are required if we want to use similar sized datasets such as those available for 18S rDNA sequences for haemogregarines, especially in terms of vertebrate host diversity (amphibians, reptiles, fishes, birds and mammals) and geographical distribution.

This study highlights the importance of screening anurans from different families and genera in an effort to increase the known biodiversity of these parasites and types of hosts they infect. This study also shows the significance of providing detailed descriptions or reports of species, localities and host records, as we were able to link a species reported by Ball (1967) with *H. thori* n. sp. in the current study based on the morphological details he provided. However, although morphological details are important, the use of them in combination with molecular tools provides a richer dataset with which to work, allowing us to infer historical relationships. Furthermore, if molecular data were available for all the currently recognized species of *Hepatozoon*, those with close morphological characteristics could be correctly distinguished. This stresses the importance of using both of these techniques in combination when describing species, and where possible to provide molecular data for already described species. Future research should, when possible, include faster-evolving genes, identification of possible definitive hosts or vectors and life cycle studies.
